# Contraceptive Use Affects Overall Olfactory Performance: Investigation of Estradiol Dosage and Duration of Intake

**DOI:** 10.1371/journal.pone.0167520

**Published:** 2016-12-21

**Authors:** Kathrin Kollndorfer, Iris Ohrenberger, Veronika Schöpf

**Affiliations:** 1 Department of Biomedical Imaging and Image-guided Therapy, Medical University of Vienna, Vienna, Austria; 2 Institute of Psychology, University of Graz, Graz, Austria; 3 BioTechMed, Graz, Austria; Monell Chemical Senses Center, UNITED STATES

## Abstract

The influence of female sex steroids on cognitive performance and sensory perception has been investigated for decades. However, previous research that studied olfaction revealed inconsistent results. The main aim of this study was to investigate the effects of different ethinyl estradiol (EE) concentrations of oral contraceptives and duration of intake on olfactory function. Forty-two healthy women, with regular intake of either high or low EE dosage over at least one year and up to 15 years participated in this study. Results revealed a significant concordance between *a priori* categorization in the two groups with high and low EE dosage and data-driven hierarchical clustering (p = 0.008). Furthermore, significantly higher olfactory performance was observed in women using low-dose products compared to women using high-dosed products (p = 0.019). These findings indicate different effects of pill use with regard to EE concentration. We therefore strongly recommend the acquisition of information about EE dosage of oral contraceptives to reduce potential confounding factors when investigating sensory systems.

## Introduction

Olfactory dysfunction affects about 20% of the general population [1,2] and is associated with a decrease in the quality of life [3,4], an extended risk for the development of mental disorders [5], and many other effects on nutritional health and personal safety [6,7]. Although several previous studies have indicated a significant role of olfactory perception in everyday life, factors affecting human odor perception in the healthy population have been rarely investigated. Previous investigations focused on gender differences (for review see [8]) and reported that women outperform men in olfactory tasks [9,10]. Most of the studies claimed a female superiority, attributed to hormonal factors, which was thought to be the result of better verbal abilities regarding olfactory testing and ratings [11]. Regardless of the verbal components and olfactory test construction, basic chemosensory processing was shown to be different across sexes in direct recordings using olfactory event related potentials [12] and at a neuronal level [13]. Hormonal factors that influence sensory perception in women include normal hormonal fluctuations within the menstrual cycle, as well as the active controlling of the menstrual cycle using contraceptives. In the US alone, oral contraceptives (OCs) are used by 62% of women who use any kind of contraceptives [14–16] and their impact on women’s sensory perception are factors worth considering when investigating healthy populations, because OC intake the natural fluctuations of ovarian steroids across the menstrual cycle is manipulated. More precisely, OCs alter estradiol concentration (E2), the most bioactive and potent part of natural estrogen in women, through the administration of the synthetic steroid ethinyl estradiol (EE). This controlled manipulation of the natural female cycle has already been shown to evoke effects on the visual sense, by affecting women’s attractiveness ratings, resulting in preferring less masculine faces during OC intake [17]. However, altered preferences were found not only with regard to the visual system. Normally menstruating women prefer the body odors of men with a dissimilar genetic variant in the major histocompatibility complex (MHC), whereas pill users tend to prefer MHC similarity [18]. Although hormonal status may affect olfactory performance (for review see [19]), only very few studies have investigated the effect of OC intake on human olfaction. Caruso et al. [20] detected altered odor thresholds in women even after a three month period of intake. In contrast, a recently published study by Renfro et al. [21] suggested that hormonal status affected olfaction in women only for musk-like odors, which are related to human sexual behavior. In a previous study by our workgroup, we observed a significant positive correlation between olfactory performance and duration of OC intake, even when corrected for age of subjects [22]. This finding indicates specific effects with regard to the duration of OC intake but did not discriminate between different types of oral contraceptives. This current study was, therefore, designed to investigate how different EE concentrations of OCs had an influence on olfactory performance. Furthermore, we investigated if the duration of OC intake had an influence on olfactory measures based on EE dosage.

## Materials and Methods

### Subjects

Fifty healthy women who regularly took oral contraceptives participated in this cross-sectional study. Data on OC intake were collected retrospectively. Four subjects had to be excluded from the data set due to incomplete measurements. An additional four subjects had to be excluded due to inconsistencies in OC intake. The final study sample consisted of 42 women, between 18 and 34 years of age (mean age, 23.8 years; standard deviation [SD] 3.8 years). All participants had no history of mental illness or neurological disorders, and they were not taking any medication known to interfere with olfactory performance. Subjects had no history of former pregnancy. Only women with regular OC intake over at least one year were included in this study. All subjects had to use the same OC product for the duration of intake or, if different products were used, these products had to have exactly the same dosing of EE and the additional substance. All women were using monophasic products; subjects using extended cycle products were excluded. For detailed information on the products see supporting information (S1 Table). Forty regularly menstruating women without any OC intake served as control condition. Olfactory performance of these women was assessed across different phases of the menstrual cycle, in order to form a representative sample of non-users of OCs. Raw data were taken from a previous data set of our study group, published in Derntl et al. [22]. The study was approved by the Ethics Committee of the Medical University of Vienna and was conducted in accordance with the Declaration of Helsinki (1964). All subjects were informed about the aim of the study and gave their written informed consent prior to inclusion.

### Olfactory performance

Olfactory performance was assessed using the Sniffin’ Sticks test battery (Burghart Instruments, Wedel, Germany). This clinically approved test battery consists of three subtests that evaluate nasal chemosensory function: odor detection threshold; odor discrimination ability; and odor identification ability. All odors of this test battery were presented using pen-like devices [23–25]. The odor detection threshold was assessed using *n*-butanol in a single-staircase, three-alternative, forced-choice procedure. The second test, which evaluated odor discrimination ability, consisted of 16 triplets of odors, in which two pens contained the same odor and the third pen contained an odd odor. All participants were instructed to detect the odd one out in each triplet of odors in a forced-choice procedure. To assess odor identification ability, subjects were presented with 16 common odors, which had to be identified using a multiple-choice answering format with a list of four descriptors for each odor. For the odor detection threshold, scores range from 1–16, for the other two subtests, scores from 0–16 may be achieved. The results of all three subtests were summed to obtain a measure of olfactory performance, the TDI (Threshold-Detection-Identification) score; thus, TDI scores ranged from 1–48.

### Statistical analysis

The vast majority of currently available OC products in Austria contain 0.020 or 0.030mg EE. Only a few products contain 0.035mg. These products are rarely prescribed to younger females, usually only in cases where a specific antiandrogenic effects were desired, e.g. skin or hair conditions. In order to avoid a bias with respect to the women’s age, we decided to separate the whole study sample in two groups: low-dose EE (LOW), with a dosage of 0.020mg EE; and high-dose EE (HIGH), with a dosage of 0.030mg EE. Subjects with higher-dosed products were excluded. This resulted in a sample size of 20 women in the LOW group and 22 women in the HIGH group.

To verify whether this division into subgroups based on EE dosage amount also corresponded to a natural division of groups in the data set, a cluster analysis was performed. To identify possible clusters in the data set independent of previous group assignments (HIGH, LOW) agglomerative hierarchical cluster analysis was performed using the Statistics Toolbox as implemented in MATLAB (Release 2014b, The MathWorks, Inc., Natick, Massachusetts, US). Similarity measures between every pair of objects (duration of intake and TDI scores) were calculated and a hierarchical cluster tree was built by using the distance information. Clustering was subsequently conducted by pruning the branches of the hierarchical tree based on the consistency criterion.

Further statistical analysis was performed using the Statistical Package for the Social Sciences (SPSS, Chicago, Illinois, USA), version 20.0. Statistical power analysis was performed using the statistical program G*Power (http://www.gpower.hhu.de/). For the detection of small to medium effects (f = 0.32) in one-way ANOVA with α = 0.05 and an assumed power (1-β) = 0.80, a total sample size of 80 subjects was calculated. In a next step, an interrater reliability was calculated, using the statistical measure of Cohen’s Kappa, in order to verify the concordance between the results of clustering and EE doses.

For all test scores, mean and standard deviation (SD) were calculated. All variables fulfilled the requirements for parametric testing. Thus, group comparisons between the two dosing levels and the control group of women who did not use OCs were computed using an analysis of variance (ANOVA). In order to gain a deeper insight into the impact of the duration of OC intake, a regression analysis was performed for the HIGH and LOW EE dose groups separately. The alpha level for all statistical tests was set to α = 0.05.

## Results

Mean duration of oral contraceptive intake was 5.68 years (LOW: 5.98 years, HIGH: 5.35 years). TDI scores ranged from 32.75 to 48 (mean 39.19, SD 3.19). Detailed results of all subtests are presented in Table 1. No significant group differences were observed in age or the mean duration of intake. However, the three groups differed significantly in their overall olfactory performance (mean TDI scores (SD): LOW 40.27 (3.28); HIGH 38.00 (2.68); NO OCs 36.49 (3.31), p<0.001). Women of the control group were significantly older (p = 0.013), however age was not correlated with any olfactory performance measure (TDI: r = -0.133, p = 0.233; threshold: r = -0.188, p = 0.101; discrimination: r = 0.153, p = 0.171; identification: r = -0.042, p = 0.711) and was therefore not included as a covariate in further analysis.

**Table 1:** Detailed results of olfactory performance measures in the two groups of low (0.020mg EE) and high-dose (0.030mg EE) OCs and the control condition of women who do not take any OCs.

	LOW	HIGH	NO OCs^a^	p-value^b^
	Mean (SD)	Mean (SD)	Mean (SD)	
	(n = 22)	(n = 24)	(n = 40)	
Age	23.82 (3.95)	23.80 (3.74)	27.08 (5.64)	0.013
Duration of intake (in years)	5.98 (2.69)	5.35 (3.64)	-	0.528
TDI	40.27 (3.28)	38.00 (2.68)	36.49 (3.31)	< 0.001
Threshold	13.13 (2.74)	12.05 (2.56)	9.14 (2.45)	< 0.001
Discrimination	13.23 (1.60)	12.95 (0.83)	13.73 (0.99)	0.078
Identification	13.91 (1.28)	13.00 (1.59)	13.63 (1.63)	0.147

^a^ Data of regularly menstruating women were taken from Derntl et al. [22].

^b^ p-values were only calculated for overall olfactory performance, as this score was the sum of the three subtests.

In a group comparison using a one-way ANOVA, a statistically significant main effect of group in overall olfactory performance (TDI; F(2,79) = 10.165, p<0.001, η^2^ = 0.205). Post-hoc analysis revealed that women of the LOW EE dose group achieved significantly higher TDI scores than women of the HIGH EE (p = 0.023) and the NO OCs groups (p<0.001). No differences were observed between HIGH EE and NO OCs (p = 0.084). Post-hoc analyses were corrected for multiple testing using Bonferroni correction. Statistically significant differences were observed for the threshold score (F(2,79) = 13.102, p<0.001, η^2^ = 0.335). No statistically significant differences were obtained for odor discrimination and odor identification (see Table 1).

**Fig 1:**
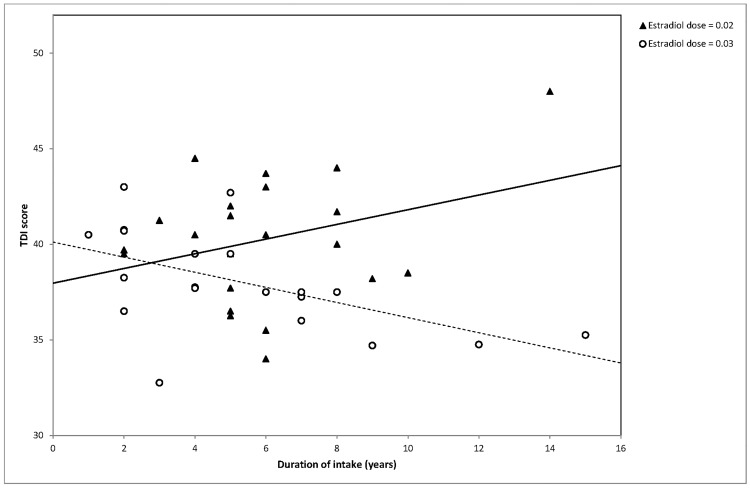
Graphic representation of the correlation between overall olfactory performance (TDI score) and the duration of OC intake. High (circles) and low (triangles) estradiol dosage in OC products are presented separately. Lines mark the corresponding linear trend. This graph shows the interaction between EE dose and duration of intake. Each point in the plot represents the individual response of a participant in the olfactory performance assessment.

Linear regression analysis revealed a significant negative association between duration of intake and olfactory performance in women who use OCs with high EE doses (R^2^ = 0.289, p = 0.014). In the low EE group no statistically significant association between duration of intake and olfactory performance was detected (R^2^ = 0.101, p = 0.150). The scatter plot in Fig 1 demonstrates the difference in overall olfactory performance measures with regard to duration of intake for the two groups.

**Fig 2:**
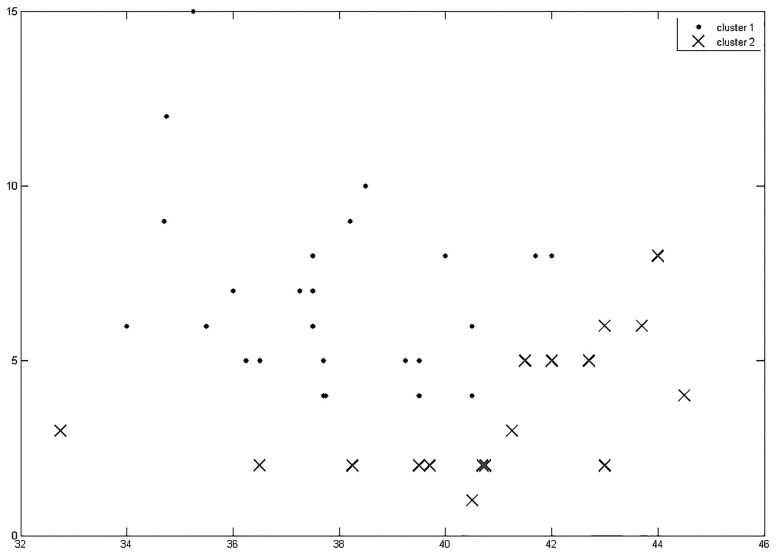
Cluster analysis. Agglomerative hierarchical cluster analysis for the whole data set to define the natural division of groups: Pairs of object were defined in the data set (for every subject: duration of intake and TDI score), then a similarity matrix representing every pair of objects of the data set was obtained and a hierarchical cluster tree was built by using the distance information. Clustering then was subsequently conducted by pruning the branches of the hierarchical tree based on the consistency criterion and two subclusters were obtained (cluster 1: marked with dots, cluster 2: marked with crosses).

Agglomerative hierarchical cluster analysis for the whole data set was performed to define a natural split within the data set by using similarity measures for every pair of objects (overall olfactory performance score and duration of intake for every subject). Based on the maximum distance criterion two clusters were thus obtained (see Fig 2).

As a measure of reliability between the categorization by clustering and the pre-assignment into the two groups LOW and HIGH, Cohen’s Kappa was calculated. Results revealed a significant concordance between the two types of categorization (Kappa = -0.410, p = 0.008). The group assignment was therefore confirmed by data-driven cluster analysis; group comparisons were performed using the differentiation LOW (0.020mg EE) and HIGH (0.030mg EE).

## Discussion

The present study aimed to investigate the effect of long-term use, as well as level of EE dose in OCs on olfactory performance, with special regard to EE dosage and duration of intake. A group comparison revealed a significantly higher overall olfactory performance in women who used products with low EE dosage (0.020mg) compared to women who used products with high EE levels (0.030mg), and to women who did not use oral contraceptives. A significant interaction between the duration of intake and olfactory performance was only observed in the high dose group but not in the low dose group. The fluctuation of female hormones throughout the menstrual cycle and its influence on cognitive performance, sensory perception and emotional states has been investigated for decades (for review see [19]). However, previous studies about the impact of fluctuating female hormones on olfactory performance provided contradictory results. Whereas most studies suggest an increased olfactory sensitivity during the ovulatory/luteal phase (e.g. [22,26,27], a time period with increased estrogen and progesterone levels in normally menstruating women, other studies did not show significant differences in olfactory sensitivity during different cycle phases [28,29]. However, Pause et al. [28] detected differences in chemosensory event-related potentials in different cycle phases. These inconsistent results might be attributable to the different odors used in the studies [29].

Previous research was also dedicated to the effect of artificial changes in female sex hormones due to OCs or hormonal replacement therapy (HRT). In this research field, the results were also contradictory. Whereas some studies provided evidence of a positive impact of HRT in postmenopausal women in various cognitive domains (for review see [30]) as well as olfactory sensitivity [31,32], there are also investigations that have shown a lack of significant differences in individuals using HRT [33]. Due to a huge increase in OC use within the last decades, numerous studies have been performed to investigate the influence of hormones in premenopausal women on various cognitive functions, such as verbal memory [34] or mental rotation [35]. However, in many studies, there were no differences found in cognitive performance between OC users and normally menstruating women (for review see [36]). The results of the present study are not in line with previous results of our working group [22], which was that women who use OCs for a longer time period, achieve higher scores in olfactory performance measures. However, the results of this study provide evidence that EE concentration level is the critical factor that was not taken into account in our previous study. Women, who use products with low EE doses (0.020mg) show higher overall olfactory performance, compared to women who used products with higher doses (0.030mg). This inter-variable effect was furthermore confirmed by the group affiliation into the high and low group by an intrinsic hierarchical clustering approach, which was based solely on the data, without previous assignment. This finding may therefore clarify previous challenges in investigations of the effects of OC intake on sensory perception.

One possible reason OCs affect olfactory performance may be an altered processing in the central nervous system (CNS), as the CNS is a major target of sex hormone action and shows a high expression of sex hormone receptors. Research results of the last decades provided evidence that estrogen is an important modulator of a broad variety of functions (for review see [37]). A manipulation of these natural targets in the CNS leads to reorganization of multifaceted network characteristics. Modifications of neural activation patterns in OC users were identified for a variety of tasks: emotional memory [38]; face recognition [39]; reward processing [40]; and even resting-state functional connectivity [41]. It has been shown that neuronal activation patterns in women who use OCs were comparable to those of men, whereas their behavioral performance was comparable to normally menstruating women [42]. In animal studies the organizational effect of prenatally administered hormones has been discovered decades ago [43]. However, we still lack knowledge about the reversibility of these changes in neural activation and the influence of synthetic EE administration. Thus, future studies could focus on the importance of the duration of intake and persistent modifications of neural activation in former OC users.

It has also been shown that estradiol treatment in mice alters the olfactory epithelium [44]. After estradiol treatment, the astrocyte density and the thickness of the olfactory epithelium was increased. Moreover, the number of mature neurons in the olfactory epithelium was increased after estradiol treatment. A study on the African cichlid fish [45] provided evidence that the olfactory bulb is a substrate for sex steroid modulation. The reproductive state regulated the expression sex steroid receptors in the olfactory bulb. The authors concluded that changes in receptor levels, may be an important mechanism for the regulation of reproductive, social and seasonal plasticity. Aromatase, the enzyme which is responsible for the conversion of androgens to estrogens, is expressed in central olfactory brain regions of rats, such as the piriform cortex [46]. More precisely, it was found that aromatase expression in the olfactory bulb was involved in the reproductive behavior in female mice [47]. These findings in animal studies provide evidence for the close interaction between estrogen receptor activity and the olfactory system.

The findings obtained in the present study raise the question of the impact of OC use on other sensory systems and cognitive functions. Although previous literature suggests that OC intake affects mating preferences (see for example [17,18]), we still lack knowledge about major influencing factors, such as duration of intake, and exact dosage of EE. Thus, future studies could focus on OC intake and the potential reversibility of alterations and interaction effects in sensory perception and cognitive function. The results of the present study did not suggest an impact of the duration of OC intake on odor perception. The results of this study suggest EE dose as the major impact factor on olfactory performance. Women who use OCs with lower EE doses (0.020mg) achieved higher olfactory performance scores compared to OC users with higher dose products (0.030mg). Interestingly, the duration of intake was associated with olfactory performance only in the high dose but not in the low dose group. Thus far, it is unclear why higher EE doses are associated with lower olfactory performance measures, which have shown to decrease with longer intake. Women who use low dosed products have significantly higher olfactory performance scores irrespective of the duration of intake. One explanation may be a negative impact on estrogen receptors, which are located in the nasal mucosa [48,49]. Female ovarian hormones and their relationship to nasal pathophysiology have already been investigated (for review see [50]). It has already been shown that OC intake may alter the nasal physiology [51]. Previous studies have already reported modified bacterial compositions in the nasal mucosa with regard to OC intake [52,53]. Recently, changes in estrogen receptor distribution in the nasal mucosa have also been observed related to the menstrual cycle phase [54] and even related to OC intake [55]. We, therefore, assume that higher EE doses in OCs negatively influence estrogen receptor regeneration cycles. As the synthetic steroid EE is added to the system during OC intake, natural endogenous estrogen production and receptor regeneration cycles may be disturbed, resulting in decreased olfactory performance after long-term use of OCs with higher EE doses. This assumption is in line with a recently published study which showed that estradiol modulated odorant responses in rodents [56]. The mechanism of estradiol impact on overall olfactory performance might therefore be the result of a two-sided inducement of CNS estradiol receptors and the microbiome of the nasal mucosa.

A possible limitation of the present study is the use of different OC products within the study sample. A broad range of products is currently available with different doses of EE as well as different additional substances such as gestagen. However, data regarding the product, and the exact dosing were collected (see S1 Table). As this study was designed as a cross-sectional retrospective study, it was not possible to acquire baseline measures before OC intake. However, we acquired a homogeneous study sample with respect to age and educational background. Another important issue is the control group. Controls groups are essential in assessing the efficacy of a treatment. However, in this research field, the definition of a control group is difficult as women underlie hormonal fluctuations throughout their menstrual cycle, which is related to differences in olfactory performance (for review see [8]). Based on these considerations, we decided to define a control group of normally menstruating women, irrespective of the menstrual cycle phase.

## Conclusion

The present study indicates an effect of EE dosage on olfactory performance measures. Subjects, who used lower dose OCs (0.020mg), achieved higher olfactory performance scores compared to women using OCs with higher EE doses (0.030mg). For clinical and behavioral studies, we therefore strongly recommend the acquisition of information about EE dosage levels of OCs to reduce possible confounding heterogeneity factors when investigating sensory systems. For female cohorts, it should be considered that including women who use different methods of (hormonal) contraception could lead to various effects on sensory thresholds. Thus, we recommend acquiring data on hormonal contraception in basic research on odor perception and in clinical studies, as this may also influence data on olfactory dysfunction.

## Supporting Information

S1 Table Detailed information on oral contraceptive products and duration of intake. (DOCX)
