# Supplementary material for: Contraceptive Use Affects Overall Olfactory Performance: Investigation of Estradiol Dosage and Duration of Intake
Source: PLoS One. 2016 Dec 21;11(12):e0167520. doi: 10.1371/journal.pone.0167520 (PMC5176159; doi:10.1371/journal.pone.0167520)
Supplement: S1 Table — (DOCX) [file pone.0167520.s001.docx]

**S1 Table.** Detailed information on oral contraceptive products and duration of intake

| ID | Product name | Ethinyl estradiol dose (mg) | Additional substance | | Duration of intake (ys) |
| --- | --- | --- | --- | --- | --- |
|  |  |  | Name | Dose (mg) |  |
| 01 | Yasminelle | 0.020 | Drospirenon | 3.0 | 6 |
| 02 | Yris mite | 0.030 | Gestoden | 0.075 | 2 |
| 03 | Yaz | 0.020 | Drospirenon | 3.0 | 3 |
| 04 | Valette | 0.030 | Dienogest | 2.0 | 12 |
| 05 | Microgynon 21 | 0.030 | Levonorgestrel | 0.15 | 4 |
| 06 | Stella | 0.030 | Dienogest | 2.0 | 3 |
| 07 | Lamuna 20 | 0.020 | Desogestrel | 0.15 | 6 |
| 08 | Yaz | 0.020 | Drospirenon | 3.0 | 4 |
| 09 | Madonella | 0.030 | Levonorgestrel | 0.15 | 2 |
| 10 | Microgynon 21 | 0.030 | Levonorgestrel | 0.15 | 1 |
| 11 | Aida | 0.020 | Drospirenon | 3.0 | 5 |
| 12 | Velafee | 0.030 | Dienogest | 2.0 | 2 |
| 13 | Valette | 0.030 | Dienogest | 2.0 | 15 |
| 14 | Yaz | 0.020 | Drospirenon | 3.0 | 4 |
| 15 | Belara | 0.030 | Chlormadinon acetat | 2.0 | 6 |
| 16 | Yirala | 0.03 | Drospirenon | 3.0 | 5 |
| 17 | Yaz | 0.020 | Drospirenon | 3.0 | 5 |
| 18 | Volina mite | 0.020 | Drospirenon | 3.0 | 5 |
| 19 | Belara | 0.030 | Chlormadinon acetat | 2.0 | 2 |
| 20 | Meliane | 0.020 | Gestoden | 0.075 | 14 |
| 21 | Valette | 0.030 | Dienogest | 2.0 | 8 |
| 22 | Yris mite | 0.030 | Gestoden | 0.075 | 7 |
| 23 | Belara | 0.030 | Chlormadinon acetat | 2.0 | 7 |
| 24 | Belara | 0.030 | Chlormadinon acetat | 2.0 | 4 |
| 25 | Belinda | 0.020 | Desogestrel | 0.15 | 10 |
| 26 | Yasmin | 0.030 | Drospirenon | 3.0 | 4 |
| 27 | Yasminelle | 0.020 | Drospirenon | 3.0 | 5 |
| 28 | Yirala | 0.030 | Drospirenon | 3.0 | 2 |
| 29 | Yasminelle | 0.020 | Drospirenon | 3.0 | 5 |
| 30 | Meliane | 0.020 | Gestoden | 0.075 | 2 |
| 31 | Loette | 0.020 | Levonorgestrel | 0.10 | 5 |
| 32 | Valette | 0.030 | Dienogest | 2.0 | 5 |
| 33 | Loette | 0.020 | Levonorgestrel | 0.1 | 2 |
| 34 | Yasminelle | 0.020 | Drospirenon | 3.0 | 6 |
| 35 | Yaz | 0.020 | Drospirenon | 3.0 | 6 |
| 36 | Yasminelle | 0.020 | Drospirenon | 3.0 | 8 |
| 37 | Selina mite | 0.020 | Levonorgestrel | 0.1 | 8 |
| 38 | Mercilon | 0.020 | Desogestrel | 0.15 | 8 |
| 39 | Maxim | 0.030 | Dienogest | 2.0 | 9 |
| 40 | Yasminelle | 0.020 | Drospirenon | 3.0 | 9 |
| 41 | Leona Hexal | 0.020 | Levonorgestrel | 0.1 | 6 |
| 42 | Madonella | 0.030 | Levonorgestrel | 0.15 | 7 |
